# Variable Fall Climate Influences Nutrient Resorption and Reserve Storage in Young Peach Trees

**DOI:** 10.3389/fpls.2018.01819

**Published:** 2018-12-17

**Authors:** Brian T. Lawrence, Juan Carlos Melgar

**Affiliations:** Department of Plant and Environmental Sciences, Clemson University, Clemson, SC, United States

**Keywords:** senescence, climate variability, mineral nutrition, water deficit, remobilization, dormancy, deciduous fruit trees

## Abstract

A delay of leaf senescence resulting from variable fall climate may allow for additional nutrient resorption, and storage within reserve organs. Autumn leaves and reserve organs (<1 year shoots, >1 year shoots, stem above and below the graft union, the tap root, and fine roots) during dormancy of young peach trees were evaluated following warmer fall temperatures and limited soil moisture on two cultivars (‘Scarletprince’ and ‘Autumnprince’ both on Guardian^TM^ rootstock) over two seasons. Four treatments were established for the two cultivars: (1) well-watered trees (100% ET_c_ needs) in ambient outdoor temperatures; (2) water deficient trees (50% ET_c_ needs) in ambient outdoor temperatures; (3) well-watered trees grown within a greenhouse; and (4) water deficient trees within a greenhouse. The greenhouse environment was on average 5°C warmer than the ambient outdoor temperature. Senescence was delayed on greenhouse-grown trees both years with leaf number and area similar in the greenhouse and outdoor environments prior to senescence. Across leaf samples, leaf nitrogen and phosphorus concentrations were lower within delayed senescence tree leaves while potassium was lower in leaves experiencing normal senescence. During dormancy, multiple reserve organs showed higher nitrogen, phosphorus, and potassium in trees with delayed senescence than normal senescence and similar increases were observed in water-deficient trees compared to well-watered trees. Phosphorus and potassium concentrations were also higher in multiple reserve organs within ‘Autumnprince’ trees compared to ‘Scarletprince’ trees. This study suggests variable climate conditions of increased temperatures or reduced soil moisture during autumn resulting in delayed senescence influence the process of nutrient resorption and increase nutrient storage within reserve organs.

## Introduction

The seasonal movement of mobile nutrients from autumn leaves to winter reserves within deciduous trees has been well documented, but variability in fall climate leading to changes in phenology could alter efficiency of nutrient resorption and preferred nutrient storage location (Titus and Kang, 1982; Estiarte and Peñuelas, 2015). Mature trees are known to efficiently remobilize nutrients, especially nitrogen (N), in comparison to immature trees (Niederholzer et al., 2001). Variability in climate (Powell and Keim, 2014; Senkbeil et al., 2017; Yu et al., 2018) could alter the movement of nutrients from annual to perennial organs and potentially impact the initial spring growth which relies on nutrient storage before mature leaves form and greater nutrient uptake from the soil occurs (Marchin et al., 2010; Jordan, 2015). Overtime, these changes of nutrient resorption between leaves and dormant organs could affect nutrient storage and how they are removed from orchards, eventually influencing annual nutrient applications (El-Jendoubi et al., 2013; Carranca et al., 2018).

Worldwide, there is an increasing delay of senescence on deciduous trees (Estiarte and Peñuelas, 2015; Yong et al., 2016). Many deciduous fruit trees initiate senescence through sensing a reduction in photoperiod and temperature (Charrier and Améglio, 2011; Naschitz et al., 2014), but the process has been shown to be slowed down following increased late-summer temperatures (Fu et al., 2018) or limited soil moisture (Naschitz et al., 2014). Depending on the plant type, resorption efficiencies vary but have been generalized as 62% for N and 65% for phosphorus (P) (Vergutz et al., 2012). Efficiency depends upon leaf and reserve nutrient status, annual temperature and moisture (Jordan et al., 2011; Vergutz et al., 2012). Potassium (K) resorption efficiency is more challenging to quantify, as the highly mobile ions can be removed through leaching, but is currently assumed to be around 70% (Vergutz et al., 2012; Estiarte and Peñuelas, 2015). Niederholzer et al. (2001) calculated that 80% of N acquired during the fall in peach trees was from leaves, revealing the importance of nutrient resorption for storage and new growth. Limited soil moisture has been shown to reduce the efficiency of N and P resorption from senescing leaves, but this depends upon the duration and timing of water deficit (Marchin et al., 2010; Khasanova et al., 2013). Alternatively, resorption of N and P can be proficient within drought-deciduous tree species and deemed complete, even under extreme drought conditions (Killingbeck, 1996; Marchin et al., 2010). Other influences on resorption include pests and pathogens which disrupt internal physiology and photosynthesis (Landeros et al., 2004; Cao et al., 2015; Moscatello et al., 2017) and are strongly linked to plant phenology (Peñuelas and Filella, 2001). For instance, reduced photosynthesis as a result of spider mites can hinder resorption of nutrients, as nutrient storage comes at the cost of carbohydrates (Bi et al., 2004; Landeros et al., 2004). As nutrients are returned to reserves during the time of senescence, climate disruptions preventing efficient resorption may influence nutrient availability for tree metabolism during dormancy and early spring growth (Estiarte and Peñuelas, 2015).

During dormancy, nutrient storage occurs in various organs depending on tree age, but primarily within roots in the case of young trees (Fan et al., 2010; Naschitz et al., 2014). Studies on peach and oak trees showed the highest concentration of N in close proximity to future growth, often within younger shoots (Estiarte and Peñuelas, 2015) or higher-order fine roots (Zadworny et al., 2015). In the case of variable climate which results in a delay of senescence, the sink strength of one or more nutrient pools may be preferentially favored, or have additional time to accumulate nutrients from leaf tissue, changing the amount of nutrients within perennial reserve organs prior to abscission.

Provided a delay in senescence caused by continued warm temperature and/or reduced soil moisture, we hypothesized trees would increase resorption of N, P, and K from their leaves, and thereby increase quantities of N, P, and K stored within perennial reserves compared to trees experiencing normal senescence (NS). How variable fall climate alters seasonal nutrient dynamics of young peach trees has not been explored and eventually may be used to optimize fertilization practices. Thus, the objective of this research was to explore how variable fall climate factors leading to a delay of senescence changes nutrient resorption from leaves to reserve storage within peach trees (*Prunus persica* (L.) Batsch).

## Materials and Methods

### Location, Design, and Treatments

This study took place between fall 2016 and winter 2018 at Clemson University, Clemson, SC, United States. A total of 126 2-year-old peach trees were grown in three gallon pots containing a mixture of Fafard 3B potting soil and 60 g 14–14–14 slow release fertilizer and divided into eight treatments resulting from a 2×2×2 factorial design comprised of three main factors: (1) *senescence timing*: trees were either kept adjacent to a greenhouse under a covered structure fully open to ambient outdoor fall temperatures for NS, or inside a greenhouse which averaged 5°C warmer than ambient outdoor fall temperatures for delayed senescence (DS); (2) *soil moisture*: trees were either well-watered (100% of their evapotranspirative needs; ET_c_) or water deficient (50% ET_c_); and (3) *cultivar of peach*: ‘Scarletprince’ and ‘Autumnprince’ both on Guardian^TM^ rootstock, with ‘Scarletprince’ being a mid-season cultivar and ‘Autumnprince’ a late-season cultivar. All treatments included 16 trees except for two treatments under ambient fall temperatures which received 50% ET_c_, both containing 15 trees.

Each fall, treatments began the first week of September and were maintained until the end of December, when all trees were placed together under the covered structure with ambient outdoor temperatures so that natural leaf abscission was ensured for all trees. At that time, all trees were well-watered to field capacity every 3–4 days until they lost all their leaves. In February 2017, half of the dormant trees were harvested and samples were taken for nutrient analyses while the rest remained in the covered area outside the greenhouse until September 2017, when the treatments mentioned above were consecutively applied to the same trees. Remaining trees in the spring of 2017 bloomed and produced fruitlets, which were removed between shuck-off and fruit set. A single application of 200 ppm ammonium nitrate (34–0–0) was applied during July 2017. Treatments concluded at the end of December 2017, and samples for nutrient analyses were taken again in February 2018. After the 2016 fall season, it was believed adjacent greenhouse lighting affected the decrease of day length and subsequent timing of senescence on the peach trees in both treatments. Therefore, black plastic curtains were erected to block additional light in both the ambient temperature and greenhouse environments during fall 2017. Additionally, all trees became infested with spider mites during the summer of 2017 when trees were placed together in the covered, ambient temperature environment. To reduce populations of spider mites, rotational weekly sprays of either abamectin, chlorfenapyr, fenpyroximate and/or potassium salts of fatty acids were applied according to their respective labels starting Sept 1 until total leaf abscission in both the ambient temperature and greenhouse environments.

### Leaf Nutrient Analysis

Three replications of leaf samples (comprised of 20, fully developed 4–5th node leaves from five trees) were taken from each treatment in 2016 (September 21, October 12, November 9, December 1, December 14), and two replications in 2017 (September 14, October 7, November 1, November 20, December 6) after the tree number was reduced in each treatment during 2017. Collected leaves were washed in distilled water, kiln dried for ≥72 h at 70°C, ground (≤1 mm), and homogenized. A subsample of 0.1 g from the resulting material was taken for measuring total N using a revised Dumas method (Harry and Jones, 1991). Another subsample of approximately 0.25 g was ashed at 600°C in a muffle furnace (LE4/11 RC, Nabertherm©, Lilienthal, Germany) for ≥15 h and remaining ashes were suspended in 10 mL of 0.1 M HCl and filtered at 0.42 μm. Phosphorus and K concentrations were measured from the filtered ash solution using the molybdenum blue colorimetric method (Murphy and Riley, 1962) and an atomic absorption spectrophotometer (PinAAcle 500, PerkinElmer, Waltham, MA, United States), respectively.

### Reserve Organ Nutrient Analysis

Prior to initial bud-swell at the end of dormancy, 15 g dry weight samples from most recently grown shoots (<1 year shoots), 1-year-old shoots from the previous growth cycle (>1 year shoots), stem above the graft union (stem), stem below the graft union (below graft), the large tap root (tap root), and fine roots (fine roots) were cut from trees. The sampling from each of the tree parts was as follows: a total of five to seven shoots from the previous year that were 10–20 cm long were cut excluding the top 10 cm of growth beneath the terminal buds; three to four 10–15 cm long sections were used from the center portions of 1-year prior growth shoots; one to two 5–6 cm long sections were removed 2–3 cm above and below the graft union; a 5–6 cm section was cut from the tap root 10 cm below the soil surface; and five 10–20 cm long fine roots ≤0.5 cm in diameter were collected. All root and fine root samples were washed to remove soil and then all samples were immediately dried for 2 weeks at 70°C and ground to ≤2 mm using a Wiley mill (Model 4, Thomas Scientific, Swedesboro, NJ, United States). The ground samples then followed the same procedure described for leaf nutrient analysis to measure nutrients.

### Statistical Analysis

The experiment followed a 2×2×2 factorial design from the three main factors. Reserve organ nutrient analysis had eight replicates per treatment during the spring of 2017 and four replicates during the spring of 2018. The effects of treatment factors and their interactions on nutrient concentrations of leaves in fall and reserve organs in winter were studied using analysis of variance (ANOVA). Student’s least significant difference (LSD) mean separation test was used to analyze differences between leaf nutrient concentrations on sample dates and between treatment means of reserve organs. Tukey’s honest significant difference (HSD) mean separation test analyzed differences of treatment interactions. Data were analyzed using JMP^®^ software (Version 12.2.0; SAS Institute, Cary, NC, United States).

## Results

### Leaf Nutrients

**FIGURE 1:**
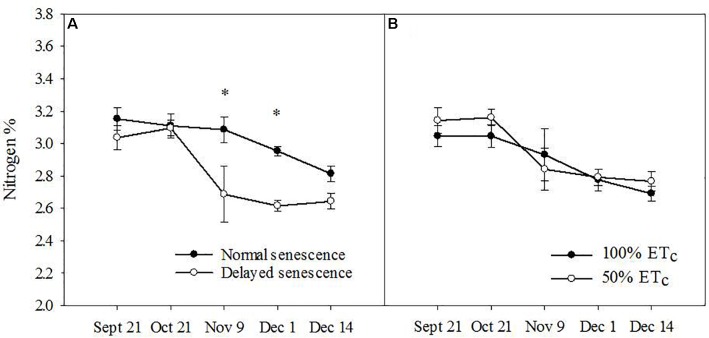
Leaf nitrogen percentage for normal senescence trees (filled circles) and delayed senescence trees (empty circles) **(A)** and soil moisture of well-irrigated trees (100% daily evapotranspirative needs [100% ET_c_], filled circles) or water deficient trees (50% ET_c_, empty circles) **(B)** during the fall of 2016. Asterisks show statistical differences (α = 0.05) between the two treatments at a given date using Student’s LSD test.

Across the fall season of 2016, leaf N was higher in NS trees (*F* = 12.86, *P* ≤ 0.01) in comparison to DS trees (Figure 1A), but not during the fall of 2017, where a triple interaction occurred between all three main factors (N concentration increased in NS ‘Autumnprince’ trees as a consequence of water deficit irrigation but not in NS ‘Scarletprince’ trees; Table 1). There were no differences in leaf N between the two soil moisture treatments (Figure 1B), nor between the two cultivars in 2016 (data not shown).

**Table 1:** Triple interaction between the three main factors of senescence timing (normal senescence or delayed senescence), soil moisture (100% ET_c_ or 50% ET_c_), and cultivar (‘Autumnprince’ or ‘Scarletprince’) on leaf N percentages during the fall of 2017.

Interaction	*% N*
Normal senescence × 100% ET_c_ × ’Autumnprince′	1.75 d
Normal senescence × 100% ET_c_ × ’Scarletprince′	2.23 ab
Normal senescence × 50% ET_c_ × ’Autumnprince′	2.24 a
Normal senescence × 50% ET_c_ × ’Scarletprince′	2.09 abc
Delayed senescence × 100% ET_c_ × ’Autumnprince′	1.8 cd
Delayed senescence × 100% ET_c_ × ’Scarletprince′	1.9 bcd
Delayed senescence × 50% ET_c_ × ’Autumnprince′	2.09 abc
Delayed senescence × 50% ET_c_ × ’Scarletprince′	2.02 abcd


*^*a*^Letters identify significant differences using Tukey’s HSD mean separation test (α = 0.05).*

**FIGURE 2:**
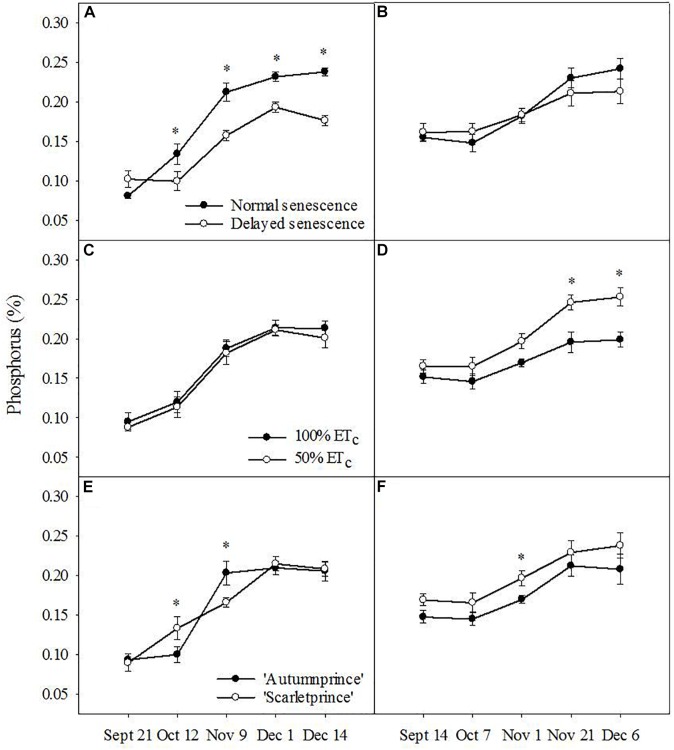
Leaf phosphorus percentage across dates by the main factors of senescence: normal senescence (filled circles) or delayed senescence (empty circles) during the fall of 2016 **(A)** and 2017 **(B)**; soil moisture: well-irrigated (100% daily evapotranspirative needs [100% ET_c_], filled circles) or water deficient (50% ET_c_, empty circles) during the fall of 2016 **(C)** and 2017 **(D)**; and cultivar: ‘Autumnprince’ (filled circles) or ‘Scarletprince’ (empty circles) during the fall of 2016 **(E)** and 2017 **(F)**. Asterisks show statistical differences (α = 0.05) between the two treatments at a given date using Student’s LSD test.

Trends of P resorption were inconsistent between the 2 years of study. DS trees showed less leaf P than NS trees as fall progressed in 2016 (*F* = 8.67, *P* ≤ 0.01), but there was no difference in 2017 (Figures 2A,B). Likewise, no consistent differences were observed between the two soil moisture treatments or cultivars in 2016 (Figures 2C,E, respectively), but 100% ET_c_ trees showed lower leaf P than 50% ET_c_ trees (*F* = 12.97, *P* ≤ 0.001) and ‘Autumnprince’ trees tended to have lower leaf P than ‘Scarletprince’ trees (*F* = 6.28, *P* ≤ 0.01) in 2017 (Figures 2D,F, respectively).

**FIGURE 3:**
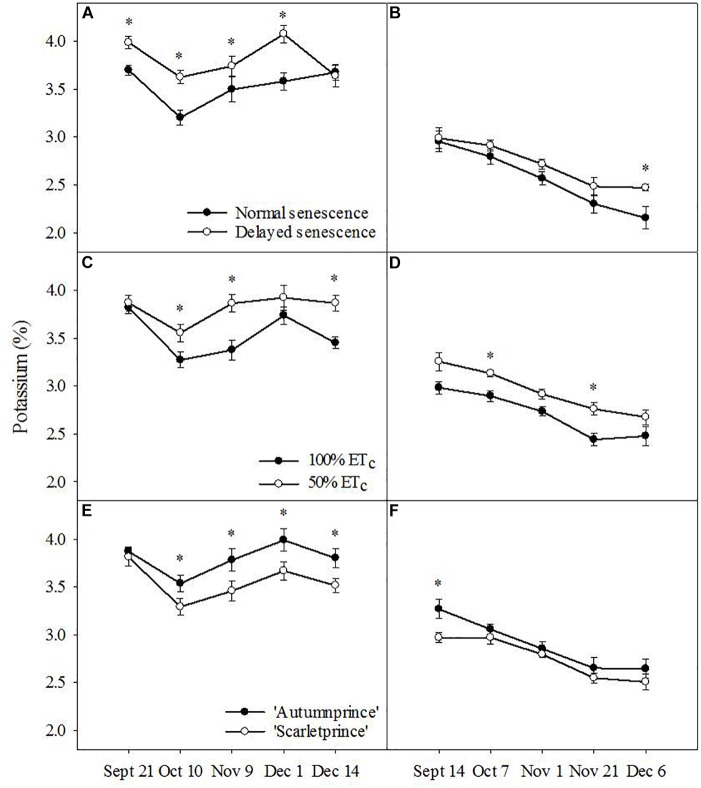
Leaf potassium percentage across dates by the main factors of senescence: normal senescence (filled circles) or delayed senescence (empty circles) during the fall of 2016 **(A)** and 2017 **(B)**; soil moisture: well-irrigated (100% daily evapotranspirative needs [100% ET_c_], filled circles) or water deficient (50% ET_c_, empty circles) during the fall of 2016 **(C)** and 2017 **(D)**; and cultivar: ‘Autumnprince’ (filled circles) or ‘Scarletprince’ (empty circles) during the fall of 2016 **(E)** and 2017 **(F)**. Asterisks show statistical differences (α = 0.05) between the two treatments at a given date using Student’s LSD test.

Unlike N and P, leaf K was higher in DS trees than NS trees (*F* = 25.94, *P* ≤ 0.001) across the fall season in 2016 (Figure 3A). The following year, DS trees had only one date of higher leaf K concentrations than NS trees at the end of 2017 (Figure 3B). Leaf K concentrations were higher in 50% ET_c_ trees across the fall of 2016 (*F* = 26.03, *P* ≤ 0.001) (Figure 3C) and 2017 (*F* = 16.92, *P* ≤ 0.001) (Figure 3D). Additionally, leaf K concentration was higher in ‘Autumnprince’ trees in comparison to ‘Scarletprince’ trees in both 2016 (*F* = 19.74, *P* ≤ 0.001) (Figure 3E) and 2017 (*F* = 5.12, *P* ≤ 0.05) (Figure 3F).

### Nutrients in Reserve Organs

Across all organ locations sampled in 2017, N concentration was significantly higher within DS trees (*F* = 10.67, *P* ≤ 0.01) and trees which had 50% ET_c_ (*F* = 5.52, *P* ≤ 0.05), but there were no differences between cultivars. The concentration of P was statistically higher in trees irrigated with 50% ET_c_ (*F* = 6.05, *P* ≤ 0.05), but not significant for the other two main factors. Concentrations of K were significantly higher in DS trees (*F* = 16.85, *P* ≤ 0.001) compared to NS trees, and in ‘Autumnprince’ trees compared to ‘Scarletprince’ trees (*F* = 4.89, *P* ≤ 0.05), but no significant differences were found across all organ locations regarding soil moisture.

Examination of individual organ locations during dormancy in 2017 following the fall of 2016 revealed significant differences based upon senescence, soil moisture, and cultivar (Table 2). A delay of senescence resulted in much higher concentrations of N, P, and K in multiple organ locations in comparison to NS trees, while the same elements appeared more concentrated in trees which were applied 50% ET_c_ in the fall compared to 100% ET_c_. Similar individual locations appeared to be affected as well, with both the >1 year shoots and below graft organ samples showing higher N, P, and K concentrations in DS trees than NS trees, and higher concentrations in 50% ET_c_ trees than in 100% ET_c_ trees. Nutrients also appeared to segregate by either roots or shoots, with P and K being higher within shoot organs in 50% ET_c_ trees compared to 100% ET_c_ trees. Trends regarding the cultivar treatment showed higher K concentrations for ‘Autumnprince’ trees than ‘Scarletprince’ trees.

**Table 2:** Percent nutrient concentrations (%) of peach tree organs during dormancy in 2017 following three main factors applied the fall of 2016: timing of senescence (a delay of senescence [DS], or normal senescence [NS]), soil moisture (providing all daily evapotranspiration requirement at 100% ETc [100%] or half at 50% ETc [50%]), and cultivar (‘Autumnprince’ [Ap] and ‘Scarletprince’ [Sp]).

Factor and location	Nutrient analyzed					
	Nitrogen (%)		Phosphorus (%)		Potassium (%)	
Senescence	DS	NS	DS	NS	DS	NS
<1 year shoots	^a^1.87^∗∗∗^	1.57	N.S.	N.S.	N.S.	N.S.
>1 year shoots	0.97^∗∗^	0.85	0.14^∗∗∗^	0.12	0.95^∗∗∗^	0.78
Stem	N.S.	N.S.	N.S.	N.S.	0.62^∗∗∗^	0.39
Below graft	0.92^∗∗∗^	0.73	0.13^∗∗∗^	0.11	0.27^∗∗∗^	0.20
Tap root	1.77^∗∗∗^	1.39	N.S.	N.S.	N.S.	N.S.
Fine roots	N.S.	N.S.	N.S.	N.S.	0.96^∗∗^	0.79

**Soil moisture**	**100%**	**50%**	**100%**	**50%**	**100%**	**50%**

<1 year shoots	1.63	1.80^∗^	0.18	0.22^∗∗^	1.13	1.25^∗∗^
>1 year shoots	0.86	0.95^∗^	0.12	0.14^∗∗∗^	0.82	0.91^∗∗^
Stem	N.S.	N.S.	N.S.	N.S.	0.48	0.54^∗∗^
Below graft	0.76	0.89^∗∗∗^	0.12	0.13^∗^	0.22	0.25^∗∗^
Tap root	1.48	1.68^∗^	N.S.	N.S.	N.S.	N.S.
Fine roots	2.27	2.64^∗∗∗^	N.S.	N.S.	N.S.	N.S.

**Cultivar**	**Ap**	**Sp**	**Ap**	**Sp**	**Ap**	**Sp**

<1 year shoots	N.S.	N.S.	N.S.	N.S.	N.S.	N.S.
>1 year shoots	N.S.	N.S.	0.14^∗∗∗^	0.12	0.93^∗∗∗^	0.80
Stem	N.S.	N.S.	N.S.	N.S.	0.57^∗∗∗^	0.45
Below graft	N.S.	N.S.	N.S.	N.S.	0.25^∗∗^	0.22
Tap root	N.S.	N.S.	N.S.	N.S.	N.S.	N.S.
Fine roots	N.S.	N.S.	N.S.	N.S.	N.S.	N.S.


*^*a*^Result of LSD analysis; N.S., non-significant; ^∗^*P* ≤ 0.05; ^∗∗^*P* ≤ 0.01; ^∗∗∗^*P* ≤ 0.001.*

There were several significant interactions in 2017, with each nutrient analyzed resulting in at least one interaction with a particular reserve location (Table 3). Within tree stems, N was lower in ‘Scarletprince’ trees which were water deficient, and higher in DS trees which were well-watered. Phosphorus was lower within fine roots of NS trees which were water deficient compared to well-watered, DS trees. Shoots of ‘Autumnprince’ trees which had a delay of senescence had much higher K than either ‘Scarletprince’ trees or NS trees.

**Table 3:** Significant interactions between main factors on nitrogen (N), phosphorus (P), and potassium (K) concentration percentages (%) in organ locations during dormancy in 2017.

Plant
organ	Type of interaction	Nutrient analyzed		
		*N (%)*	*P (%)*	*K (%)*
*>1 year*				
*shoots*
	Normal senescence × ’Autumnprince′			^a^1.08 bc
	Normal senescence × ’Scarletprince′			0.98 c
	Delayed senescence × ’Autumnprince′			1.52 a
	Delayed senescence × ’Scarletprince′			1.18 b
*Stem*				
	100% ET_c_ × ’Autumnprince′	0.62 b		
	100% ET_c_ × ’Scarletprince′	0.65 ab		
	50% ET_c_ × ’Autumnprince′	0.76 a		
	50% ET_c_ × ’Scarletprince′	0.64 ab		
				
	Normal senescence × 100% ET_c_	0.62 b		
	Normal senescence × 50% ET_c_	0.60 b		
	Delayed senescence × 100% ET_c_	0.80 a		
	Delayed senescence × 50% ET_c_	0.66 b		
*Fine roots*				
	Normal senescence × 100% ET_c_		0.16 ab	
	Normal senescence × 50% ET_c_		0.14 b	
	Delayed senescence × 100% ET_c_		0.19 a	
	Delayed senescence × 50% ET_c_		0.17 ab	


*^*a*^Letters identify significant differences using Tukey’s HSD mean separation test (α = 0.05).*

The following year, dormant tree organ analyses revealed N was not significant across organ locations for senescence timing, soil moisture, and cultivar. Phosphorus was not different based on senescence, but significantly higher in 50% ET_c_ trees (*F* = 3.99, *P* ≤ 0.05) than in 100% ET_c_, and in ‘Autumnprince’ trees (*F* = 4.03, *P* ≤ 0.05) than in ‘Scarletprince’ trees across organ locations. Potassium was higher across organ locations in DS trees (*F* = 3.91, *P* ≤ 0.05), but similar in soil moisture and cultivar treatments.

Analysis of nutrient concentrations within individual tree organ locations in 2018 showed similar trends compared to the results in 2017, but there were no differences in N concentration between DS and NS trees, with only the shoots of NS trees showing higher N concentrations than DS trees (Table 4). Several dormant organs (<1 year shoots, below graft, and tap roots) of 50% ET_c_ trees again showed higher N concentrations than 100% ET_c_ trees. Also, P and K revealed significant differences regarding senescence and soil moisture for multiple locations, specifically higher concentrations of both nutrients in DS trees compared to NS trees, and higher concentrations of P for <1 year shoots, >1 year shoots, stem and below graft samples of 50% ET_c_ trees compared to 100% ET_c_ trees. Nevertheless, trends within specific organ locations such as >1 year shoots or below graft union were less consistent than in 2017. The cultivar treatment had a greater influence on nutrient differences in 2018 than 2017, especially for P, which showed higher concentrations in above-ground organs of ‘Autumnprince’ than in ‘Scarletprince.’

**Table 4:** Percent nutrient concentrations (%) of peach tree organs during dormancy in 2018 following three main factors applied the fall of 2017: timing of senescence (a delay of senescence [DS], or normal senescence [NS]), soil moisture (providing all daily evapotranspiration requirement at 100% ET_c_ [100%] or half at 50% ET_c_ [50%]), and cultivar (‘Autumnprince’ [Ap] and ‘Scarletprince’ [Sp]).

Factor and organ	Nutrient analyzed					
	Nitrogen (%)		Phosphorus (%)		Potassium (%)	
*Senescence*	DS	NS	DS	NS	DS	NS
<1 year shoots	0.86	^a^0.99^∗^	0.21^∗∗^	0.19	N.S.	N.S.
>1 year shoots	N.S.	N.S.	0.17^∗∗∗^	0.15	0.42^∗∗∗^	0.37
Stem	N.S.	N.S.	0.13^∗∗^	0.11	N.S.	N.S.
Below graft	N.S.	N.S.	N.S.	N.S.	0.35^∗^	0.31
Tap root	N.S.	N.S.	N.S.	N.S.	N.S.	N.S.
Fine roots	N.S.	N.S.	N.S.	N.S.	0.58^∗^	0.53

***Soil moisture***	**100%**	**50%**	**100%**	**50%**	**100%**	**50%**

<1 year shoots	0.87	0.98^∗^	0.18	0.22^∗∗∗^	N.S.	N.S.
>1 year shoots	N.S.	N.S.	0.14	0.17^∗∗∗^	N.S.	N.S.
Stem	N.S.	N.S.	0.11	0.13^∗∗^	N.S.	N.S.
Below graft	0.57	0.68^∗∗^	0.08	0.09^∗^	N.S.	N.S.
Tap root	1.02	1.2^∗∗^	N.S.	N.S.	N.S.	N.S.
Fine roots	N.S.	N.S.	N.S.	N.S.	0.51	0.60^∗∗^

***Cultivar***	**Ap**	**Sp**	**Ap**	**Sp**	**Ap**	**Sp**

<1 year shoots	N.S.	N.S.	0.21^∗^	0.19	N.S.	N.S.
>1 year shoots	N.S.	N.S.	0.18^∗∗∗^	0.14	0.42	0.38^∗∗^
Stem	N.S.	N.S.	0.13^∗∗∗^	0.10	N.S.	N.S.
Below graft	N.S.	N.S.	N.S.	N.S.	N.S.	N.S.
Tap root	1.18^∗^	1.04	0.11	0.13^∗^	N.S.	N.S.
Fine roots	N.S.	N.S.	N.S.	N.S.	N.S.	N.S.


*^*a*^Result of LSD analysis; N.S., non-significant; ^∗^*P* ≤ 0.05; ^∗∗^*P* ≤ 0.01; ^∗∗∗^*P* ≤ 0.001.*

Only two reserve locations had statistical interactions present in 2018 (Table 5). DS trees had lower N concentration within stems than NS trees when well-watered, and ‘Autumnprince’ trees had higher P concentration in shoots than ‘Scarletprince’ trees under the water deficit treatment.

**Table 5:** Significant interactions between main factors on nitrogen (N) and potassium (K) concentration percentages (%) in organ locations during dormancy in 2018.

Plant organ	Type of interaction	Nutrient analyzed	
		*N (%)*	*K (%)*
*>1 year shoots*			
	100% ET_c_ ×′Autumnprince′		^a^0.60 ab
	50% ET_c_ ×′Autumnprince′		0.68 a
	100% ET_c_ ×′Scarletprince′		0.64 ab
	50% ET_c_ ×′Scarletprince′		0.59 b
*Stem*			
	Normal senescence × 100% ET_c_	0.60 a	
	Normal senescence × 50% ET_c_	0.59 a	
	Delayed senescence × 100% ET_c_	0.50 b	
	Delayed senescence × 50% ET_c_	0.60 a	


*^*a*^Letters identify significant differences using Tukey’s HSD mean separation test (α = 0.05).*

## Discussion

### Effect of Senescence Timing on Nitrogen

Autumn climate conditions favoring increased temperatures that delay senescence and reduced soil moisture increased nutrient storage in perennial reserve organs, although this was not always directly related to leaf nutrient resorption patterns the previous fall. Different patterns of N storage were observed between the two years. The increase of N within DS reserve organs compared to NS organs in 2017 (not found in 2018) could be due to different timing of leaf senescence and abscission which occurred later in the fall in 2016 than the fall of 2017 allowing for thorough scouring of N. Reserve storage of N is known to be primarily accumulated from leaf resorption (Titus and Kang, 1982) as N uptake during the fall and throughout dormancy is limited (Jordan et al., 2011). As leaf number and area were similar between DS and NS trees, higher concentrations within dormant organs in 2017 could also be a result of relocating N among the perennial reserves (Gomez and Faurobert, 2002) but with the triple interaction and without differences across dormant organs in 2018, resorption during the fall of 2017 was most likely influenced by several factors including temperature, photoperiod, biotic factors, and nutrient status. Temperature influences senescence (Fu et al., 2018), and the greenhouse environment in 2017 had cooler temperatures on average than in 2016. The DS trees in 2017 began the process of senescence and abscission earlier, which could prevent full N resorption (Estiarte and Peñuelas, 2015) in comparison to 2016, as leaf attachment persisted longer into the fall. The slower senescence in 2016 may have also been due to longer photoperiod as a result of adjacent greenhouse lighting in 2016 (Charrier and Améglio, 2011). Additional lighting may have delayed the initiation of senescence and allowed for the senescence process to be generally slower, allowing trees ample time to thoroughly gather leaf N prior to abscission (Milla et al., 2005; Estiarte and Peñuelas, 2015). When curtains were erected in 2017 to prevent artificial light, senescence and abscission for both DS and NS trees occurred earlier than the previous year, but DS trees was still delayed in comparison to NS trees, with any gain from this additional attachment time not reflected for N concentrations within the perennial reserves. Additionally, there was an infestation of spider mites in fall 2017 that may have prevented trees from efficient resorption of N. Mites could have accelerated the senescence process, increased water stress, and disrupted normal source and sink relationships (Landeros et al., 2004; Estiarte and Peñuelas, 2015; Moscatello et al., 2017). A reduction of photosynthesis the second year from spider mites may have additionally reduced the amount of carbohydrates partitioned to the roots during the growing season, reducing root exploration and N uptake (Jordan et al., 1998). Much lower leaf N concentrations were observed across treatments during the fall of 2017 and could be a result of annual leaching and gaseous losses from the containers, dilution of samples via larger size trees the second year, and removal of sample leaves and fruitlets known to contain high quantities of N (El-Jendoubi et al., 2013). Despite the lower nutrient levels of both DS and NS leaves in 2017 compared to 2016, no increase of resorption was observed within the leaf analysis nor translated to perennial organs (Vergutz et al., 2012; Yuan and Chen, 2015). Other studies have also shown that leaf nutrient status does not determine resorption (Aerts, 1996) and is not consistent between years (Killingbeck, 1996). Further studies should explore the influence of nutrient status, especially since many fruit orchards are often well-fertilized regardless of variable climate conditions.

### Effect of Senescence Timing on Phosphorus

Phosphorus concentration within dormant organs was found to follow a similar trend both years, with DS trees having higher P than NS trees in multiple perennial organs, notably within above-ground sampling locations. Concentration differences of P between the DS and NS dormant organs occurred regardless of resorption differences measured in the fall, as DS trees had lower concentration of leaf P than NS in 2016 and all trees had similar concentrations in 2017. Greater concentrations of P within DS tree <1 year shoots, >1 year shoots and stems compared to NS trees in 2018 appear to support P accumulation within shoot and stem tissues (Rennenberg and Herschbach, 2013; Kurita et al., 2017). Since leaf P was similar across the fall season of 2017 however, mechanisms other than leaf resorption may have accounted for differences within organs during the winter of 2018, underscoring the need to better understand P accumulation within storage pools during different seasons (Rennenberg and Herschbach, 2013). It is assumed that deciduous trees return around 65% of leaf P return to reserves (Vergutz et al., 2012) and even for evergreen trees, as reported for citrus, root absorption during the spring is not the primary source of P, with trees relying more on stored P for new growth (Bachiega Zambrosi et al., 2012). A decrease of leaf P should occur over time during senescence (Marschner, 1995), but the increase observed both fall seasons is thought to be due to sampling method. Actively growing peach trees had prolonged shoot expansion and new leaves during the fall of 2016, but not during the fall of 2017. Consistent sampling of leaves associated with the fourth and fifth nodes from shoot termini may have inaccurately captured N and P status over the fall as the new shoots and leaves were assumed to be active sinks (Bachiega Zambrosi et al., 2012). Even without actively growing shoots in 2017, both NS and DS trees maintained leaves for a longer time at shoot termini where sampling occurred. Spider mite damage also restricted new growth in the summer and fall of 2017 (Landeros et al., 2004) and sampling of the fourth or fifth node leaves were therefore older, having fully formed during the summer months as opposed to any new shoot growth occurring during the fall. Greater amounts of P within perennial above-ground organs in DS trees the second season could also be a result of pest presence, as has been reported for huánglóngbìng infected citrus, with pathogens influencing P movement to other perennial organs (Cao et al., 2015). Since P cycling and acquisition from roots in trees is still largely unexplored (Rennenberg and Herschbach, 2013), further studies addressing resorption of P when a delay of senescence occurs should address the nutrient status prior to, during, and after senescence treatments.

### Effect of Senescence Timing on Potassium

The concentration of K also increased within dormant organs following a delay of senescence. Unlike leaf N or P, leaf K was consistently higher in DS trees than in NS trees during 2016. Leaf K concentrations were statistically similar across the fall of 2017, with DS trees having numerically higher leaf K than NS trees. Higher temperatures within the greenhouse environment may have caused an increase of leaf K concentration in order to maintain cellular function and avoid heat stress (Hasanuzzaman et al., 2018). Discrepancies between the 2 years suggest higher concentrations within DS dormant organs may have resulted not from increased efficiency of resorption, but other mechanisms of acquisition. Increased root exploration occurs within warmer soils (Pregitzer et al., 2000), and greater uptake of K may have occurred for DS trees compared to NS trees due to the warmer temperatures in the greenhouse. Potassium ions are regarded as highly mobile, and therefore the lack of leaf K concentration decrease across the 2016 fall season was unexpected, as reports of resorption are around 70% (Marschner, 1995; Vergutz et al., 2012; Estiarte and Peñuelas, 2015). Differences in K resorption observed could be attributed to a later senescence time for NS and DS trees in 2016 in comparison to 2017 or the tree nutritional status (Vergutz et al., 2012) as leaf K concentration may have been generally high within tree tissue during the fall of 2016 following a single season within the containers. As trees were not fertilized with a source of K after beginning the study in summer of 2016, the lower percentages of leaf K measured in 2017 could be attributed to leaves lost during natural abscission or from sampling, fruitlets removed, dilution as trees grew larger the second year, and general leaching known to occur from leaves and containers (Vergutz et al., 2012; El-Jendoubi et al., 2013; Estiarte and Peñuelas, 2015). Since K helps support cell function during stress events (Hasanuzzaman et al., 2018), the higher temperatures during the fall of 2016 may have encouraged greater amounts of K to be allocated to leaf tissue to assist with the resorption of N and P during the more gradual senescence period (Milla et al., 2005). Potassium removed from fallen leaves for this study alone was estimated to be between 1.5 and 2.6 g per tree during the fall of 2016, which is congruent with El-Jendoubi et al. (2013). Although export of K from leaf tissue is known to occur during senescence (Marschner, 1995), the amount remobilized to perennial organs within young peach trees may be less than hypothesized. Gradual senescence within woody plants as a result of dry conditions have shown to decrease the resorption efficiency of K, but not necessarily for N and P (Milla et al., 2005). Thus, the efficiency of K resorption within fruit trees needs further analysis using both leaf tissue and dormant organs as indicators of tree status.

### Effect of Soil Moisture on Nutrient Resorption

The water deficit treatment consistently increased nutrient concentrations within multiple perennial organs. Even with similar leaf number and area on NS trees (data not shown), and fewer leaves on water deficient, DS trees compared to well-watered DS trees, the perennial organs showed higher concentrations of N, P, and K in the 50% ET_c_ trees. These results are supported by multiple studies which have concluded water deficient conditions naturally reduce plant nutrient uptake (see He and Dijkstra, 2014 for a meta-analysis). A reduced nutrient uptake may have led to a strong nutrient resorption in water deficient plants as suggested by Zhao et al. (2017). Nevertheless, other mechanisms such as a reduction in specific leaf area in water deficient plants compared to well-watered plants could have enhanced nutrient resorption as well under conditions where nutrient uptake was reduced (Wang et al., 2017). In a similar way, decreased resorption efficiencies have been observed in grassland species during wet years in comparison to dry years (Ren et al., 2018), and resorption of amino acids decreases with greater water availability in oak trees (Suseela et al., 2015). Both Suseela et al. (2015) and Ren et al. (2018) reported the interaction of warming temperatures and reduced soil moisture greatly influenced resorption, but no interaction between soil moisture and DS due to warmer temperatures was detected for leaf sampling in our study across either of the two seasons. Several tree species deemed as drought-deciduous had complete nutrient resorption following water stress (Marchin et al., 2010) while other plants including perennial grasses show decreased efficiencies when drought occurred concurrently with autumn senescence (Khasanova et al., 2013). Without limiting resorption due to drought conditions (Marchin et al., 2010), young peach trees receiving 50% ET_c_ may increase resorption efficiencies due to increased levels of leaf tissue degradation (Suseela et al., 2015) evidenced by increased nutrient concentrations in perennial organs.

The effect of soil moisture on nutrient resorption depended on the nutrient. Both leaf N and P were not different across moisture treatments in 2016, but P did show differences in 2017, with 100% ET_c_ trees showing less leaf P than 50% ET_c_ trees. Regardless, there was an increase of reserve N and P in both years, mainly segregated to increases within the rootstock and scion, respectively. Infestation of spider mites was thought to be one reason why differences in leaf P occurred between the soil moisture treatments in 2017, by which additional moisture stress due to increased transpiration and stomatal conductance (Landeros et al., 2004) limited P resorption in the 50% ET_c_ trees (data not shown). Similarly, leaf K concentration was consistently higher in 50% ET_c_ trees in 2016 and 2017, revealing translocation of K to leaf tissue to maintain cellular function (Hasanuzzaman et al., 2018). Although much K was lost through leaf senescence during the fall of 2016, K was numerically higher in 50% ET_c_ trees across all dormant organ locations, and significantly so for several reserve locations in both 2017 and 2018, suggesting K may have been acquired through roots in addition to leaf resorption. Significantly higher levels of K within fine roots in 2018 suggests water deficient trees translocated K to increase root surface area (Hasanuzzaman et al., 2018). With the presence of spider mites, uptake of soil K may have been limited in 2017, resulting in no differences between 100% and 50% ET_c_ tree perennial reserves. Examination of root to shoot ratios showed no differences between 50% and 100% ET_c_ trees in 2016 (0.47 and 0.48, respectively) or 2017 (0.76 and 0.78, respectively), although higher 2017 values suggested shoot growth was less proficient and/or root growth was greater in 2017.

### Effect of Cultivar on Nutrient Resorption

The cultivar also influenced resorption of K and P in 2016 and 2017 and subsequent concentrations within dormant organs in 2017 and 2018, respectively. Cultivar alone did not affect the amount of N and no differences were found across dormant organs although a two-way and triple interaction showed an influence on N by cultivar. The trees used in this study were juvenile and did not have fruit (Tartachnyk and Blanke, 2004), therefore without influence on nutrient partitioning due to seasonal sinks, scion genetics alone seemed to influence resorption (Carranca et al., 2018). Nevertheless, further studies with other cultivars may provide evidence of consistent nutrient storage patterns as a result of variable fall climate. Provided these storage trends, growers could possibly optimize fertilization according to specific cultivar nutritional requirements.

## Conclusion

In summary, variable fall climate conditions which caused a delay of senescence appear to change the timing and resorption efficiency compared to NS which often resulted in higher concentrations in the dormant organs. Moderate water limitation did not always change resorption patterns, but also led to an increase of nutrients stored within reserves. Likewise, the cultivars showed differences in leaf nutrient and later reserve organ concentration. Overall, the results from the reserve organs provided a better understanding than fall leaf analysis on how the treatments affected the dynamics of nutrient movement. Inconsistencies of resorption efficiencies based upon plant nutrient status (Yuan and Chen, 2015) suggest further studies need to examine resorption within specific environments and how increasing frequency of variable climate conditions (Yu et al., 2018) alter nutrient recycling. Specifically, experiments using mature fruit trees under field conditions could explore how variable fall climate conditions affect nutrient dynamics throughout the year before drawing any conclusions on the applicability of these findings in tree orchards.

## Author Contributions

BL and JM planned and carried out the experiments together. BL took the lead in writing the manuscript with support from JM.

## Conflict of Interest Statement

The authors declare that the research was conducted in the absence of any commercial or financial relationships that could be construed as a potential conflict of interest.
